# A Dimer for Dinner: The Impact of GHS-R1a Heterodimerization on Feeding Circuits

**DOI:** 10.3390/biom16060788

**Published:** 2026-05-27

**Authors:** Tingting Tang, Qingli Zhang, Tingting Song, Dan Ding, Dejiu Zhang, Yan Zhang, Zichu Zhao, Jingjing Kong, Qu Chen, Lei Zhu, Hailong Li

**Affiliations:** Department of Medicine, Qingdao Binhai University, 425 West Jialing River Rd., Qingdao 266555, China

**Keywords:** growth hormone-releasing hormone receptor 1a (GHS-R1a), heterodimer, G protein-coupled receptors (GPCRs), signal transduction, feeding circuit, energy homeostasis

## Abstract

Growth hormone-releasing hormone receptor 1a (GHS-R1a) is a key G protein-coupled receptor (GPCR) governing feeding and energy homeostasis. Accumulating evidence shows that GHS-R1a forms functional heterodimers with multiple metabolic-related GPCRs, including dopamine 2 receptor (D2R), melanocortin 3 receptor (MC3R), 5-hydroxytryptamine 2c receptor (5-HT2cR), orexin receptor 1 (OX1R) and cannabinoid receptor 1 (CB1R). These heterodimers undergo distinct signal transduction reprogramming, generating novel physiological effects that are not observed with individual receptors: for instance, GHS-R1a/D2R mediates an atypical calcium signaling pathway to regulate appetite, while GHS-R1a/5-HT2cR antagonizes ghrelin-induced orexigenic effects. Meanwhile, diverse detection techniques, including co-immunoprecipitation and fluorescence resonance energy transfer, have been developed to identify and validate GHS-R1a heterodimerization, laying a solid foundation for mechanistic research. This review systematically summarizes the molecular mechanisms of GHS-R1a heterodimer formation, the characteristic signal regulation patterns of different heterodimers, and their specific regulatory roles in feeding circuits. Furthermore, we discuss the existing research gaps in this field, such as the lack of in vivo detection methods for heterodimers and the unclear structural basis of dimerization. Finally, we highlight the potential of targeting specific GHS-R1a heterodimers as a novel therapeutic strategy for obesity and anorexia, providing new directions for future pharmaceutical development and clinical translation.

## 1. Introduction

G protein-coupled receptors (GPCRs) constitute the largest family of transmembrane receptors [1,2]. They possess seven α-helical transmembrane domains and can form heterotrimeric complexes with G proteins, thereby regulating key physiological processes such as metabolism, signal transduction and neuronal activity [3,4,5,6,7]. In recent decades, the traditional view that GPCRs primarily exist as monomers has been re-evaluated; substantial evidence confirms that many GPCRs can form stable dimeric complexes [8,9,10], playing an increasingly significant role in the treatment of a wide range of diseases, including psychiatric disorders, cardiovascular diseases, metabolic disorders, cancer and inflammation [8,9,10].

The growth hormone-releasing hormone receptor 1a (GHS-R1a) is a typical GPCR comprising 366 amino acid residues and containing seven-pass transmembrane domain (7TM). It primarily couples to Gαq to activate classical downstream signaling pathways [11]. It is widely distributed throughout the central nervous system, exhibiting high expression not only in the hypothalamus and pituitary gland but also in the substantia nigra, cortex, hippocampus and ventral tegmental area (VTA) [12,13]. Furthermore, low levels of GHS-R1a expression have been detected in other peripheral organs such as the pancreas, spleen, kidneys and adrenal glands [14,15]. The biological functions of GHS-R1a, mediated through its binding to the ligand ghrelin, have long been a subject of widespread interest [16]. Ghrelin is a potent neuropeptide, comprising 28 amino acids, primarily secreted by X/A-like cells in the gastric fundus, with GHS-R1a acting as its functional receptor [17,18,19]. Upon activation of GHS-R1a by ghrelin, it plays a role in various biological processes, including cardioprotection, enhancement of learning and memory, regulation of feeding behavior, and promotion of growth and development [20,21,22,23]. In addition to ligand-dependent activation, GHS-R1a also exhibits high constitutive activity independent of ghrelin [24,25,26], which is mediated by transmembrane domain (TM) VI and TM VII [27,28]. In investigating the mechanisms underlying this constitutive activity, researchers found that, in the absence of agonists, TM VI and VII of GHS-R1a form an aromatic cluster through spatial proximity. Simultaneously, under the influence of salt bridges, they displace towards TM III to complete helix–helix docking. This, combined with interactions between amino acid residues within TM III, collectively stabilizes the conformation of the receptor’s spontaneous activation [24,29,30,31]. Furthermore, it has been observed that extracellular loop (ECL) 2 maintains the flexible spatial structure of GHS-R1a to ensure normal movement of the transmembrane domains; mutations in this structure result in the formation of a rigid α-helix that restricts the movement of TM V, disrupting the intrinsic activated conformation and causing the loss of intrinsic activity [32,33].

GHS-R1a can form functional heterodimers with various GPCRs involved in energy metabolism, such as the melanocortin 3 receptor(MC3R) [34,35,36], 5-hydroxytryptamine 2C receptor (5-HT2cR) [37,38,39,40], orexin 1 receptor (OX1R) [41], G protein-coupled receptor 83(Gpr83) [42], dopamine 2 receptor (D2R) [43,44,45,46,47] and the cannabinoid receptor 1 (CB1R) [48]. This mode of assembly confers novel biological properties on the receptors that differ from those of the monomers. Among these, the GHS-R1a/MC3R heterodimer produces an asymmetric signaling interference effect, significantly blocking ghrelin-induced Ca^2+^ influx and upregulating MC3R-mediated cyclic adenosine monophosphate (cAMP) signaling, thereby inhibiting the body’s appetite-stimulating signaling pathways [36,38,49]; the GHS-R1a/OX1R heterodimer, on the other hand, induces a G protein subtype shift, switching from Gαq-mediated signaling to Gαs-mediated signaling, thereby mediating the activation of downstream cAMP pathways and regulating neuronal proliferation [41]. Furthermore, different types of dimers exhibit distinct functional antagonistic and synergistic characteristics: GHS-R1a/5-HT2cR specifically inhibits GHS-R1a-dependent Ca^2+^ signaling, producing an appetite-suppressing antagonistic effect [37,38,39,40]; whereas GHS-R1a/CB1R synergistically amplifies Ca^2+^ signal intensity, further enhancing ghrelin’s appetite-stimulating action [48]. Concurrently, Gpr83 and D2R can also bind to GHS-R1a to form heterodimers, which attenuate receptor sensitivity through subunit interactions, thereby negatively regulating feeding and metabolism [42,43,44,45,46]. Furthermore, dysregulation of GHS-R1a heterodimerization is associated with metabolic disorders, making it a promising therapeutic target [50].

In summary, various GHS-R1a heterodimers regulate animal feeding behavior bidirectionally through distinct signal remodeling mechanisms, collectively participating in the fine-tuning of central energy homeostasis. Currently, research on GHS-R1a heterodimers is hampered by a lack of in vivo detection techniques and the incomplete elucidation of structural mechanisms. This paper systematically summarizes the molecular characteristics, signal regulation patterns and physiological functions of GHS-R1a dimers, aiming to provide a theoretical framework for the development of targeted drugs for diseases associated with metabolic disorders. 

## 2. GPCR Heterodimerization

### 2.1. Structural and Functional Characteristics of GPCRs

GPCRs share a common structural architecture characterized by seven TM α-helices (TM1–TM7), an extracellular N-terminus, three ECLs (ECL1–ECL3), three to four intracellular loops (ICL) (ICL1–ICL4), and an intracellular C-terminus [51,52]. The intracellular loops and C-terminus of GPCRs interact with heterotrimeric G proteins. Upon ligand binding, GPCRs undergo conformational changes that activate the coupled G protein, initiating distinct downstream signaling cascades to elicit diverse biological effects [51,52,53,54].

### 2.2. Classical GPCR-G Protein Signaling Mechanism

According to the canonical model, ligand-activated GPCRs undergo a conformational shift, enabling them to couple with a specific heterotrimeric G protein [55]. The G protein consists of α, β, and γ subunits. Receptor activation induces the exchange of guanosine diphosphate (GDP) for guanosine triphosphate (GTP) on the Gα subunit, triggering the dissociation of Gα from the Gβγ dimer [56]. The activated Gα and free Gβγ subunits then regulate distinct downstream effector systems to propagate the ligand-induced signal. Signal termination occurs when the intrinsic GTPase activity of Gα hydrolyzes GTP to GDP, allowing Gα to reassociate with Gβγ and reform the inactive heterotrimeric complex, thereby resetting the system for subsequent signaling cycles [57,58].

### 2.3. Functional Roles of G Protein Subunits

The Gα subunit serves as the primary mediator of signal transduction, whereas Gβγ primarily modulates downstream signaling regulation [59,60]. Gα proteins are classified into four major subfamilies: Gαs, Gαi/o, Gαq and Gα12/13 [61,62]. Specifically, GHS-R1a activates downstream signaling pathways predominantly through Gαq coupling [63].

The conventional view was that GPCR mostly existed and functioned as a monomer [64], until Agnati et al. proposed the possibility of mutual transformation between receptors in 1980 [65]. Later, various GPCR protein molecules that had extensive cross-talk were found, indicating that GPCR might exist in the form of structural or functional complexes. GPCR dimerization has attracted more and more attention in recent years.

Studies have shown that GPCRs interact with each other, and different subtypes and types of GPCRs can form homologous or heterogenic aggregates (oligomeric dimers and polymers, etc.) [66], resulting in the formation of basic functional units. This is not an accidental or random process, but a universally recognized physiological mechanism.

## 3. Dimerization Detection Technology

**Figure 1:**
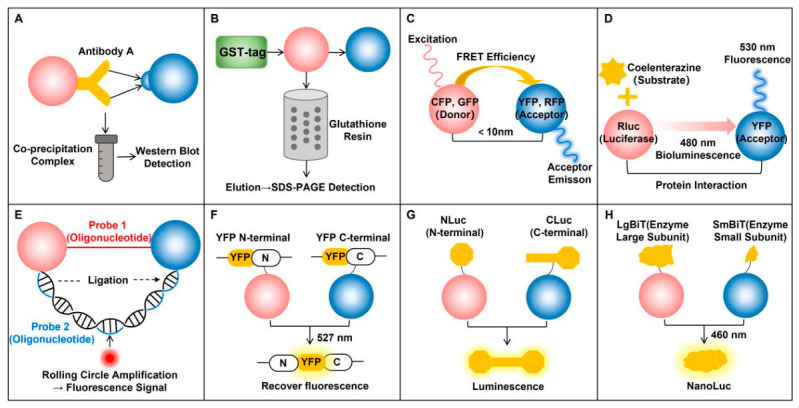
Detection techniques for GHS-R1a heterodimerization. Schematic illustration of methods for identifying and monitoring GHS-R1a heterodimerization. Protein A (red) and Protein B (blue) represent two interacting receptors in the dimer complex. (**A**) Co-IP; (**B**) GST pull-down; (**C**) FRET; (**D**) BRET; (**E**) PLA; (**F**) BiFC; (**G**) BiLC; (**H**) NanoBiT.

Over the past two decades, a number of techniques and methods for detecting protein interactions have been developed, such as the commonly used co-immunoprecipitation (Co-IP) [67,68], Glutathione-s-transferase (GST) pull-down assay [69], proximity ligation assay (PLA) [70], fluorescence resonance energy transfer (FRET) [71], bioluminescent resonance energy transfer (BRET) [72], bimolecular fluorescence complementation (BiFC) [73], bimolecular luminescence complementation (BiLC) [74,75,76], and nanoLuc binary technology (NanoBiT) [77] protein complementary techniques [78] (Figure 1). These methodologies can be broadly categorized based on their experimental systems: in vitro approaches utilizing cell lysates (Co-IP and GST pull-down assays) and in vivo live-cell imaging techniques (PLA, BRET, FRET, BiFC, BiLC, and NanoBiT).

The Co-IP assay is a well-established biochemical technique designed to validate protein–protein interactions between two known proteins (Protein A and Protein B). In this assay, Protein A is immunoprecipitated using a specific antibody conjugated to Protein A/G-coated agarose beads. Subsequent Western blot analysis is then performed to detect the presence of co-precipitated Protein B [79] (Figure 1A). Reciprocally, the interaction can be confirmed by reversing the immunoprecipitation–Western blot strategy. This bidirectional Co-IP approach provides definitive experimental evidence for the physical interaction between Protein A and Protein B [77,80].

The GST pull-down assay represents an in vitro affinity purification technique that shares conceptual similarities with Co-IP in investigating protein–protein interactions, but differs fundamentally in its experimental approach [81]. Unlike Co-IP, which examines endogenous protein interactions within intact cellular systems, the pull-down assay utilizes purified recombinant proteins that are genetically engineered with distinct affinity tags. In this controlled system, the tagged proteins are co-incubated in solution, followed by affinity capture of one protein using tag-specific matrices (Figure 1B). The presence of potential interacting partners is subsequently detected through Western blot analysis, thereby validating the physical association between the proteins of interest [82,83].

The FRET assay represents a highly sensitive spectroscopic technique that enables the detection of molecular interactions at nanometer-scale proximity [84,85]. This phenomenon occurs through non-radiative energy transfer between two adjacent fluorophores when the emission spectrum of the donor fluorophore significantly overlaps with the absorption spectrum of the acceptor fluorophore, and the molecular separation is within 10 nm [86]. The FRET process manifests through two characteristic optical signatures: substantial quenching of donor fluorescence intensity and marked enhancement of acceptor emission (sensitized fluorescence) (Figure 1C). This technique offers unique advantages for studying dynamic protein–protein interactions, as it permits real-time monitoring of both association and dissociation events within protein complexes that exist in dynamic equilibrium under physiological conditions [86,87]. The capacity to detect transient molecular interactions in live cells makes FRET particularly valuable for investigating the spatiotemporal regulation of protein complexes in their native biological context [88].

The BRET assay represents an advanced adaptation of FRET technology that employs a bioluminescent luciferase enzyme to replace one of the fluorescent proteins in the conventional FRET system [89,90]. Compared with FRET, BRET exhibits several distinct advantages: it eliminates the requirement for external excitation light, thereby significantly reducing background noise; it minimizes phototoxicity and cellular damage; and it provides stronger luminescent signals due to the small molecular weight of the donor NanoLuc luciferase [91] (Figure 1D). These technical improvements make BRET particularly suitable for sensitive and prolonged monitoring of molecular interactions in live cells and organisms.

The PLA employs a pair of oligonucleotide-conjugated antibodies as its core proximity probes. Following co-incubation of primary antibodies, PLA probes, and target proteins, when two target proteins are in close proximity (<40 nm), the system initiates a signal amplification cascade through sequential binding of secondary antibodies and rolling circle amplification, ultimately generating microscopically visible fluorescent spots (~1 μm in diameter) with red emission (Figure 1E). This unique design enables direct visualization of interacting protein pairs at their subcellular localization [92]. Compared to conventional techniques, PLA offers superior resolution and quantitative capabilities, making it particularly valuable for investigating protein–protein interactions, protein phosphorylation status, and protein expression levels with enhanced sensitivity and spatial precision.

BiFC is a protein-fragment complementation assay that utilizes split non-fluorescent fragments of fluorescent proteins, which are separately fused to two target proteins of interest. Upon interaction between the target proteins, the complementary fragments reconstitute into a functional fluorophore that emits detectable fluorescence upon excitation [93] (Figure 1F). This technique enables direct visualization and rapid assessment of protein–protein interactions and subcellular localization in living cells, offering high signal-to-noise ratios and the capability to detect weak interactions (>7 nm). BiLC is very similar to BiFC; the core principle of both is based on the complementarity of protein fragments, but the difference lies in the fact that the reporter protein in BiLC is luciferase [76] (Figure 1G). The NanoBiT technology shares conceptual similarities with BiFC but exhibits significant methodological improvements. The key distinction lies in their reporter systems: BiFC employs split yellow fluorescent protein (YFP) fragments [93], whereas NanoBiT utilizes split NanoLuc luciferase fragments. Due to the smaller molecular weight, stronger luminescent signal, and higher expression efficiency of NanoLuc, NanoBiT demonstrates approximately two orders of magnitude greater sensitivity compared to conventional BiFC (Figure 1H). Furthermore, the reversible nature of NanoBiT tag association enables real-time monitoring of dynamic protein–protein interactions in live cells [94]. These technological advancements in protein-fragment complementation assays, particularly the development of highly sensitive and reversible systems like NanoBiT, have been instrumental in driving the rapid progress of GPCR interaction research over the past two decades.

## 4. What Is GHS-R1a?

GHS-R1a, the endogenous receptor of ghrelin, which contains 7TMs, can bind to ghrelin and exert biological effects [95]. GHS-R1a widely expressed in different tissues, including the hippocampus, cortex, the hypothalamus, pituitary, dentate gyrus, the VTA and the substantia nigra [95,96,97,98,99,100,101]. GHS-R1a exerts a wide range of biological effects in vivo by binding to the ligand ghrelin [102].

Known as a classic GPCR, GHS-R1a mediates the major signaling pathways, including the Ca^2+^ signaling pathway, the phosphatidylinositol (PI)-specific phosphatidylase (PLC) pathway, the protein kinase C (PKC) pathway [103] and adenosine monophosphate-activated protein kinase (AMPK) signaling pathway [104]. Of course, GHS-R1a can also cascade with the signal of phosphatidylinositol 3 kinase (PI3K) to regulate Ca^2+^ channels by activating protein kinase A (PKA) in hypothalamic neurons [105]. The combination of ghrelin and GHS-R1a activates PLC, and the hydrolysis of phosphatidylinositol-4, 5-diphosphate (PIP-2) on the plasma membrane by PLC produces diacylglycerol (DAG) and inositol triphosphate 3 (IP3) [97,106]. Then, IP3 binds to receptors on the endoplasmic reticulum to promote the release of Ca^2+^ from intracellular Ca^2+^ stores, resulting in a rapid and transient increase in intracellular Ca^2+^ concentration. Meanwhile, DAG activates PKC on plasma membrane and inhibits potassium channels through tyrosine phosphorylation, leading to depolarization. Subsequent depolarization can activate voltage-dependent L-type Ca^2+^ channels and extracellular Ca^2+^ influx, resulting in a long-term increase in intracellular Ca^2+^ concentration [107,108,109]. The GHS-R1a-mediated Ca^2+^ signaling pathway not only participates in regulating excessive secretion of ghrelin in a mouse model of type 2 diabetes [110], but also, by maintaining cellular Ca^2+^ homeostasis, inhibits apoptosis in cardiomyocytes and endothelial cells and improves left ventricular function during ischemia–reperfusion injury [111], thereby exerting a cardioprotective effect [112,113], as well as increasing food intake in guinea pigs [114]. In addition to the classical PLC/Ca^2+^/PKC signaling pathway, GHS-R1a also regulates energy homeostasis via the AMPK signaling pathway, which is a core energy-sensing mechanism in hypothalamic neurons [115]. Activated AMPK acts as a key molecular switch, promoting food intake by upregulating the appetite-stimulating neuropeptides neuropeptide Y (NPY) and agouti-related peptide (AgRP) whilst simultaneously suppressing the appetite-suppressing neuropeptide (proopiomelanocortin) POMC [116,117,118]. This AMPK-dependent neuropeptide regulation is one of the core mechanisms by which GHS-R1a promotes appetite, and is particularly crucial during states of negative energy balance, such as hunger.

## 5. GHS-R1a and Feeding

**Figure 2:**
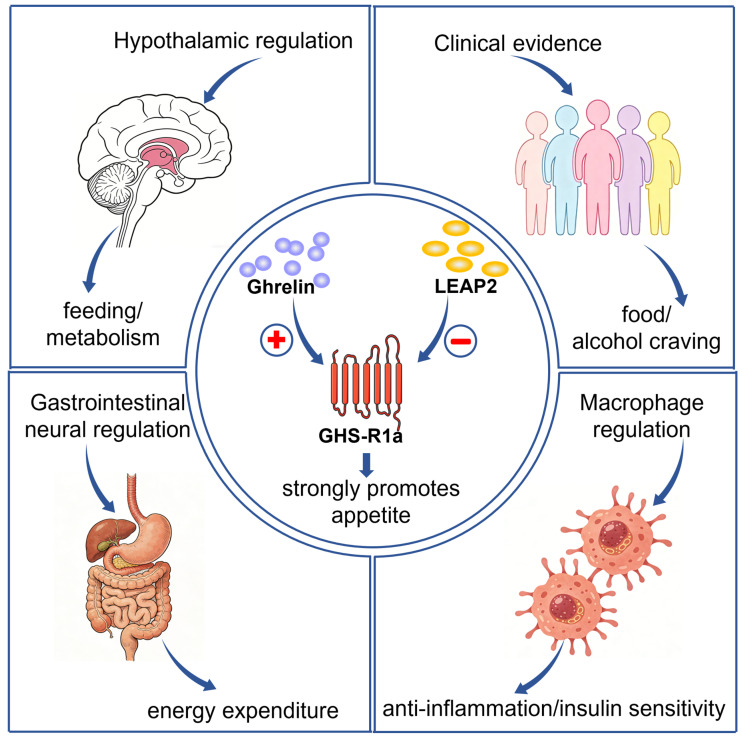
The regulatory role of GHS-R1a in appetite activation and energy homeostasis. GHS-R1a regulates feeding and metabolism via ghrelin-dependent activation and its constitutive activity in both central and peripheral tissues. It controls appetite through hypothalamic circuits and modulates energy expenditure, inflammation and insulin sensitivity in the gastrointestinal tract and macrophages. As an endogenous antagonist of GHS-R1a, LEAP2 inhibits receptor function, counteracts ghrelin-induced orexigenic and hyperglycemic effects, and together with the ghrelin–GHS–R1a axis, it maintains whole-body energy balance.

GHS-R1a is the key receptor for ghrelin; this receptor is extensively involved in the regulation of appetite and energy homeostasis and plays a significant role in metabolic regulation (Figure 2). Ghrelin is an appetite-stimulating hormone secreted by the fundus of the stomach, and the fundamental function of GHS-R1a is to regulate digestion and feeding. Furthermore, this receptor possesses intrinsic activity; even in the absence of ghrelin stimulation, its activity can still reach 50% of its maximum level [26,31,108,119,120]. In terms of energy homeostasis regulation, GHS-R1a achieves multi-tissue, multi-dimensional metabolic regulation via multiple signaling pathways: in the hypothalamus, this receptor regulates metabolism via Kisspeptin/Neurokinin B/Dynorphin (KNDy) neurons, and GHS-R1a knockout (KO) alleviates high-fat diet-induced obesity [121]. In gastrointestinal sensory neurons, GHS-R1a enhances sympathetic innervation of adipose tissue and promotes energy expenditure; knockout of this receptor improves diet-induced obesity, and this effect is independent of feeding behavior [122]. In macrophages, GHS-R1a induces inflammatory polarization and glycolysis; its knockout reduces inflammation and improves insulin resistance [123]. Concurrently, GHS-R1a expression regulates ghrelin signaling in a dose-dependent manner; heterozygous mice, despite retaining partial feeding regulation, still exhibit abnormalities such as growth retardation and impaired glucose metabolism [124]. Experiments using various gene-KO animal models have further confirmed that knocking out GHS-R1a reduces food intake and body weight and improves metabolic abnormalities induced by a high-fat diet, specifically by alleviating adipose inflammation, enhancing insulin sensitivity, and increasing brown adipose tissue content [15,101,125,126,127]. Epidemiological studies also support its clinical application; administration of PF-5190457, a GHS-R1a inverse agonist, reduces cravings for alcohol and food in humans [128]. In summary, GHS-R1a not only regulates feeding behavior via central and peripheral neural circuits [129,130,131,132], but also modulates energy allocation through immunometabolic, sympathetic and tissue-specific signaling pathways (Figure 2), making it a promising potential target for the treatment of obesity and related metabolic diseases.

Within the regulatory system of GHS-R1a, liver-expressed antimicrobial peptide 2 (LEAP2) has been identified in recent years as an important endogenous regulatory ligand that participates in the regulation of the body’s metabolic balance by acting as a target for GHS-R1a. In 2003, Krause et al. first identified LEAP2 in human blood ultrafiltrate [133]. LEAP2 belongs to the class of cationic antimicrobial peptides and was initially demonstrated to possess antimicrobial and anti-infective properties [134]. As research in the field of metabolism has progressed, the antagonistic regulatory role of LEAP2 on GHS-R1a has gradually been revealed. In 2017, Xuecai Ge et al. discovered that LEAP2 is the second endogenous ligand of GHS-R1a, and also acts as an endogenous antagonist of this receptor [135]. The manner in which LEAP2 antagonizes GHS-R1a exhibits distinct dose-dependence and is influenced by the order of administration: when LEAP2 is administered in advance, it acts as a non-competitive antagonist, whereas when it is co-administered with ghrelin, it exhibits competitive antagonism [136,137]. Animal studies indicate that GHS-R1a is the key receptor through which LEAP2 regulates blood glucose and suppresses appetite in mice; infusion of LEAP2 into mice reduces postprandial blood glucose and growth hormone levels, and decreases food intake [138]. In obese states, elevated endogenous levels of LEAP2 can antagonize the biological effects of acetylated ghrelin; conversely, reduced LEAP2 levels enhance the activity of acetylated ghrelin [139].

In summary, GHS-R1a positively mediates ghrelin’s regulation of appetite and energy metabolism, whilst LEAP2, as its endogenous antagonist, negatively inhibits receptor activity; the two act in an antagonistic yet synergistic balance to jointly maintain the body’s metabolic homeostasis.

## 6. GHS-R1a Heterodimers

Ghrelin/GHS-R1a system mediated signal transduction is involved in many physiological and pathophysiological processes. The most common function is ingestion and energy metabolism. GHS-R1a not only combines with ghrelin, but also combines with a series of other GPCRs to form heterodimers, including D2R [45,140,141], MC3R [36], 5-HT2cR [37], OX1R [41], Gpr83 [42] and CB1R [48]. Interestingly, these GPCRs are closely related to energy regulation, so will these heterodimers also participate in the regulation of diet and energy metabolism?

### 6.1. GHS-R1a/MC3R Heterodimer

As a pivotal GPCR that regulates hypothalamic energy homeostasis, MC3R selectively forms heterodimers with the GHS-R1a, thereby bidirectionally modulating downstream signaling cascades through asymmetric allosteric interference—ultimately attenuating orexigenic signaling.

As a subfamily of GPCR, MC3R is expressed in the hippocampus and other parts of the hypothalamus and activates the adenylyl cyclase (AC)/cAMP signaling pathway by coupling Gαs [142,143]. MC3R is involved in a variety of physiological functions, including cardiovascular function and energy homeostasis [144,145,146,147,148]. The MC3R endogenous ligand is mainly composed of α,β,γ-Melanocyte-stimulating hormone (α,β,γ-MSH) and adrenocorticotrophic hormone (ACTH) [142,143,149]. In 2009, Rediger et al. detected the presence of GHS-R1a/MC3R dimer in COS-7 cells and HEK293 cells by sandwich ELISA and FRET, respectively [34]. Follow-up studies in 2011 further elucidated the unique bidirectional regulatory effects of this dimer [36]. Specifically, in HEK293A cell lines co-expressing MC3R and GHS-R1a, ghrelin stimulation almost completely blocked GHS-R1a-mediated Ca^2+^ influx [38].

Conversely, in co-expressing COS-7 cell lines, administration of α-MSH increased cAMP accumulation at MC3R by over thirty-fold [36]. It has been proved that the activation of GHS-R1a/MC3R heterodimer can increase the downstream signaling pathway activated by MC3R and weaken the downstream signaling pathway activated by ghrelin. This phenomenon of asymmetric signal interference may reflect a “priority” design in energy metabolism regulation: the catabolic signals from the melanocortin system can suppress ghrelin’s pro-feeding signals.

Girardet et al. found that GHS-R1a KO mice showed strong food anticipatory activity when food intake was restricted, while MC3R KO mice and GHS-R1a and MC3R double knockout mice did not exhibit any abnormal behavior. The reason for this may be that the knockout of GHS-R1a has a compensatory effect on the animal’s body, while MC3R exactly compensates for the effect of GHS-R1a [35]. These findings reveal that the activation state of one receptor within a GPCR heterodimer can determine the functional characteristics of the other receptor. This provides a novel mechanism for understanding the synergistic role of GHS-R1a and MC3R in feeding regulation, holding significant implications for the development of drugs targeting body weight regulation.

### 6.2. GHS-R1a/D2R Heterodimer

GHS-R1a exhibits extensive colocalization with the D2R in dopaminergic neurons and selectively forms heterodimers with D2R, thereby rewiring downstream signaling networks to elicit atypical Ca^2+^ responses and coordinately regulate appetite, motor function, and emotional behavior.

D2R is widely distributed in the brain, and GHS-R1a and D2R receptors are expressed in the VTA, striatum, hippocampus, thalamus, and dopaminergic neurons in the substantia nigra [97,150,151,152,153,154,155]. D2R is mainly coupled with Gαi, which mainly inhibits AC/cAMP/PKA signaling [156,157,158]. GHS-R1a is mainly coupled with Gαq [159] and the signaling pathway is activated by PLC/DAG/PKC [97]. D2R KO rats showed obvious impaired motor function, such as spontaneous motor dysfunction, decreased motion initiation frequency [160], spontaneous hypotonia [161], participation in avoidance behavior [162], decreased ability to learn movement, and abnormal gait and posture [160]. The downregulation of GHS-R1a can lead to motor coordination dysfunction, initial dopaminergic neuron dysfunction [163] and mood disorders, such as depression-like behavior [164]. The knockout of GHS-R1a can also affect the hippocampal structural integrity, resulting in spatial memory impairment [165]. Thus, there is a high degree of overlap between the two receptors.

**Figure 3:**
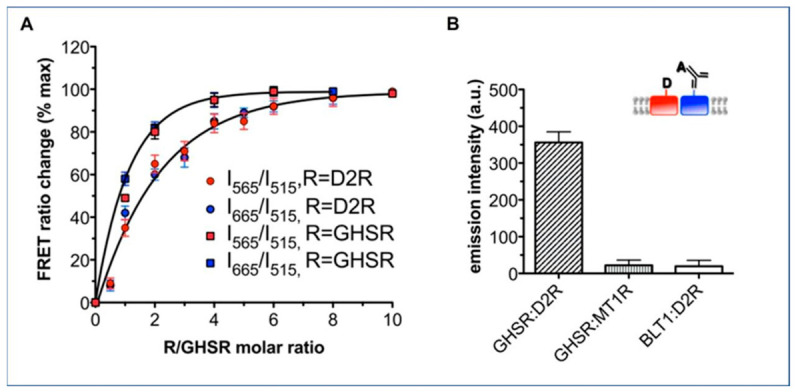
Specific heteromerization between GHS-R1a and D2R in proteoliposomes detected by FRET [141]. (**A**) The results of the FRET assay revealed a significant energy transfer signal between GHS-R1a and D2R, confirming that the two can interact directly within cells. (**B**) FRET screening revealed that GHS-R1a exhibits a strong interaction only with D2R, whilst no significant signal was observed with other GPCRs tested (such as MT1R and BLT1), highlighting the receptor specificity of this heterodimerisation. Adapted from Damian et al. [141] with permission.

Kern et al. detected the formation of GHS-R1a/D2R heterodimer in hypothalamic neurons using time-resolved FRET (tr-FRET) techniques in 2012 [43] (Figure 3). Once this dimer is formed, it triggers significant signal reorganization: D2R, which originally exerts an inhibitory effect, can activate the PLC pathway via the Gβγ subunit, inducing Ca^2+^’s release from the endoplasmic reticulum and causing the neuronal response to shift from inhibition to excitation [43]. This regulatory mechanism does not require ghrelin activation; GHS-R1a acts as an allosteric modulator, directly altering the conformation of D2R and its G protein-coupling preferences [43].

Animal studies have shown that the GHS-R1a/D2R heterodimer is essential for D2R agonist-induced anorexia. Activation of this dimer by D2R agonists produces a distinct anorexic effect; this effect is retained in ghrelin-KO mice but is completely lost in GHS-R1a-KO mice, demonstrating that the appetite-regulating function of D2R is entirely dependent on the presence of GHS-R1a [43,166].

Furthermore, the GHS-R1a/D2R heterodimer jointly regulates Ca^2+^ channel activity, influencing the firing and synaptic transmission of dopaminergic neurons, and is involved in physiological and pathological processes such as motor behavior and emotional disturbances [45]; GHS-R1a-specific antagonists can also effectively block dopamine-induced neuronal excitatory currents, further corroborating the functional coupling between the two [167].

### 6.3. The Role of the GHS-R1a/D2R Heterodimer in Regulating Neuro-Metabolic Processes

The GHS-R1a/D2R heterodimer plays a key role in the physiological and pathological regulation of feeding homeostasis, reward behavior and gastrointestinal motility, and constitutes a central functional unit in the neuro-metabolic balance mediated by the dopamine and ghrelin systems.

In terms of appetite and anorexia regulation, the GHS-R1a/D2R heterodimer plays a significant role in modulating D2R-mediated anorexia [43]. Kern et al. demonstrated that D2R agonists exert a distinct anorexic effect, and that this action is entirely dependent on GHS-R1a expression; in GHS-R1a KO mice, the anorexic effect of D2R agonists was completely abolished, and this was independent of endogenous ghrelin involvement [43]. Other studies have indicated that D2R can bidirectionally regulate the appetite-stimulating effects of ghrelin; both agonizing and antagonizing D2R can attenuate ghrelin-induced overeating behavior, suggesting that there is a complex relationship between the dopamine and ghrelin pathways in central appetite regulation [168]. Clinical studies have also confirmed that obesity can significantly alter the interaction patterns between midbrain D2R and acetylated ghrelin, disrupting normal neuroendocrine balance and thereby inducing feeding and metabolic disorders [169].

With regard to food reward and behavioral regulation, the GHS-R1a/D2R heterodimer in the VTA–nucleus accumbens pathway plays a dominant role in regulating reward motivation; ghrelin can enhance the craving for food via this heterodimer, and this behavior is dependent on D2R in the nucleus accumbens, whereas basic physiological feeding does not depend on this circuit, confirming that reward-driven feeding and ordinary feeding are characterized by distinct neural circuits [170].

In the spinal defecation center, presynaptic neurons in the lumbosacral parasympathetic ganglia co-express D2R, GHS-R1a and 5-HT2cR; dopamine can excite defecation-related neurons in a GHS-R1a-dependent manner, promoting colonic motility and the defecation reflex. Administration of a GHS-R1a antagonist significantly blocks this prokinetic effect, indicating that GHS-R1a and D2R are the key target for regulating gastrointestinal motility [167]. Interestingly, the expression of GHS-R1a/D2R also exhibits marked sex-specificity. In rats from an early-stage obesity model combined with social stress, male rats were more prone to concurrent downregulation of hypothalamic GHS-R1a and D2R, whereas female rats were relatively less affected [171].

In summary, the GHS-R1a/D2R heterodimer not only plays a central role in the regulation of feeding, reward, gastrointestinal and visceral sensation, but also exhibits significant sex-specific expression patterns; it is a key functional complex that links the dopamine and ghrelin systems and maintains the body’s neuro-metabolic homeostasis.

### 6.4. GHS-R1a/5-HT2cR Heterodimer

Both GHS-R1a and 5-HT2cR serve as core regulators of appetite and energy homeostasis; their heterodimerization selectively inhibits GHS-R1a mediated Ca^2+^ signaling and establishes an antagonistic balance between orexigenic and anorexigenic pathways.

It is well known that GHS-R1a is the endogenous receptor of ghrelin, which plays an important role in the steady-state control of food intake and energy balance [172,173,174]. Another GPCR, the Serotonin 2c receptor, also known as 5-HT2cR, has also been shown to be critically important in regulating appetite and satiety [175,176]. 5-HT2cR is mainly coupled to G_q_/G_11_-protein and it can be activated by phospholipase C [177]. It is also associated with intracellular Ca^2+^ and K^+^ flows [178]. It is noteworthy that although these two receptors belong to different GPCR families, they both occupy a central position in the regulation of energy metabolism, suggesting that they may jointly maintain metabolic homeostasis through synergistic or antagonistic interactions. Knocking out the 5-HT2c gene changed the diet of the mice, leading to excess weight and symptoms such as cognitive deficits in epilepsy [179,180]. This also suggests that the two receptors are antagonistic to each other in the regulation of diet [181].

Schellekens et al. detected the co-localization and interaction of GHS-R1a and 5-HT2cR in 2013, and GHS-R1a-induced intracellular Ca^2+^ release decreased after ghrelin or MK0677 were given to HEK293 cells that were co-expressed by GHS-R1a and 5-HT2cR [38]. In 2015, they demonstrated a direct interaction between the GHS-R1a and the 5-HT2cR via flow cytometry fluorescence resonance energy transfer (fcFRET). They found that when the 5-HT2cR signal was blocked, ghrelin-induced appetite increased. In contrast, ghrelin’s appetite-promoting function was also blocked when mice were given a 5-HT2cR agonist [37]. This pharmacological experiment directly supports the antagonistic relationship between the two receptors, demonstrating that activation of 5-HT2cR suppresses the pro-appetitive effects of GHS-R1a, thereby providing novel insights for developing anti-obesity drugs targeting the dimer. Regarding the signaling mechanism, studies have revealed that GHS-R1a and 5-HT2cR form heterodimers in HEK293 cells. Upon administration of varying concentrations of ghrelin or the agonist MK0677, GHS-R1a-mediated intracellular Ca^2+^ release exhibited a 30–80% reduction. Similarly, when the dimer was co-stimulated with 5-HT or ghrelin, or with both 5-HT and ghrelin, no changes in cAMP were observed. These results suggest that activation of the GHS-R1a/5-HT2cR dimer inhibits GHS-R1a-mediated intracellular Ca^2+^ signaling while leaving the cAMP pathway unaffected. This indicates that GHS-R1a/5-HT2cR dimerization may achieve functional antagonism between GHS-R1a and 5-HT2cR by selectively regulating specific downstream pathways. This pathway-specific modulation provides crucial insights into understanding receptor cross-talk mechanisms [37].

The primary mechanism by which the second-generation antipsychotic olanzapine induces obesity is believed to involve its antagonism of the 5-HT2cR and activation of the GHS-R1a dimer [39]. By antagonizing 5-HT2cR, olanzapine inhibits its interaction with GHS-R1a, thereby activating downstream GHS-R1a signaling pathways and increasing NPY expression. This represents a key neuro-molecular mechanism that is potentially responsible for its obesity-inducing effects. This discovery provides a potential intervention target for preventing antipsychotic-induced metabolic side effects. Drugs targeting this mechanism, such as those confirmed by research demonstrating the interaction between 5-HT2cR and GHS-R1a in hypothalamic neurons, may offer therapeutic opportunities [40].

To some extent, this indicates that the activation of GHS-R1a-mediated signaling pathway is reduced by the activation of GHS-R1a/5-HT2cR heterodimer, and the generation of intracellular Ca^2+^ is also reduced, thus regulating the functions of GHS-R1a and 5-HT2cR. The antagonistic interaction between GHS-R1a (orexigenic) and 5-HT2cR (anorexigenic) suggests dimerization as a bidirectional switch for appetite control. Overall, the dimerization of GHS-R1a with 5-HT2cR represents a novel receptor regulatory mechanism, achieving precise appetite control through physical interactions and signal interference. Future research may further explore the structural characteristics of the dimer and its therapeutic potential in metabolic disorders.

### 6.5. GHS-R1a/Gpr83 Heterodimer

As an orphan class A GPCR, Gpr83 forms heterodimers with GHS-R1a, thereby reducing GHS-R1a’s sensitivity to ghrelin and attenuating ghrelin-induced orexigenic responses; this interaction establishes Gpr83 as an essential negative regulator of energy metabolism.

**Figure 4:**
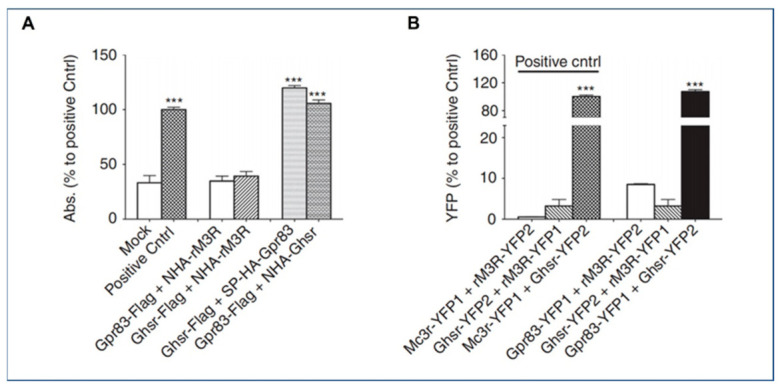
GHS-R1a forms heterodimers with Gpr83 [42]. (**A**) Sandwich ELISA experiments demonstrated that GHS-R1a and Gpr83 interact in vitro. Using a specific receptor pair as the positive control and co-transfection of unrelated receptors as the negative control, the results showed that the signal indicating interaction between the two was significantly higher than that of the control groups. (**B**) Receptor-receptor interactions were further validated using a YFP-based protein complementation assay (YFP-PCA), and the results similarly confirmed that Gpr83 and Ghsr1a form heterodimers within cells. Data represent mean ± s.e.m. *** *p* < 0.001 vs. control group. Adapted from Müller et al. [42] with permission.

The formation of GHS-R1a/Gpr83 heterodimers was first identified in 2013 [42] (Figure 4). Mouse Gpr83 is an orphan receptor [42]. Similarly to GHS-R1a, both belong to the class A GPCR family [42,182]. Gpr83 is mainly distributed in hypothalamic nucleus, such as arcuate nucleus, paraventricular nucleus and lateral hypothalamic area, which is closely related to the control of energy balance [183]. While GHS-R1a and Gpr83 were found to form a heterodimer, the team also found that the expression of Gpr83 in lean mice was decreased in vivo, and in vitro, they found that the activation of GHS-R1a was reduced due to the heterodimerization of Gpr83 and GHS-R1a. Moreover, the ghrelin-induced appetite-promoting function was more significant in Gpr83 knockout mice; that is to say, GHS-R1a/Gpr83 heterodimer can antagonize ghrelin’s appetite-promoting effect.

GHS-R1a/Gpr83 heterodimer is competitive with the ghrelin/GHS-R1a system, which is another angle for appetite control or weight control, and may provide a new angle for the research and development of weight-loss drugs or anorexia drugs.

### 6.6. GHS-R1a/OX1R Heterodimer

GHS-R1a stably forms heterodimers with OX1R, thereby inducing a switch in G protein coupling from Gαq to Gαs; this heteromeric complex regulates neuronal proliferation and integrates central feeding behavior with peripheral satiety signals.

In 2018, GHS-R1a/OX1R heterodimers have been reported to form in HEK293 cells through BRET and FRET and Co-IP [41]. The endogenous ligand Orexin-A [184] for OX1R can activate Gαq [184,185] whilst also triggering multiple downstream signaling pathways via Gαi and Gαs [186,187,188]. OX1R is widely distributed in the central nervous system, in which the locus coeruleus is highly expressed, and the medial ganglion nucleus, VTA and other brain regions are also distributed [184]. The different distribution of these brain regions may be related to different physiological functions.

In HEK293 cells that co-expressed GHS-R1a and OX1R, the stimulation of ghrelin increased intracellular cAMP accumulation, while orexin-A did not, indicating that ghrelin could induce cAMP response element signals through the GHS-R1a/OX1R heterodimer; GHS-R1a/OX1R dimer activated by ghrelin can also promote the proliferation of SH-SY5Y cells [41]. The mechanism is that ghrelin changes the original signal transduction of OX1R or GHS-R1a by activating GHS-R1a/OX1R dimer, inducing Gαs signal transduction instead of Gαi or Gαq signal transduction, and thus increasing the level of cAMP [41]. Co-activation of GHS-R1a/OX1R shifts signaling from Gαq to Gαs, promoting hippocampal neuronal proliferation; this is indicative of a potential link between feeding and memory.

In addition, recent research by Suarez et al. found that the ghrelin/GHS-R1a signal in the ventral hippocampus can be transmitted to the lateral dorsal tegmental nucleus through downstream orexin signals, thereby canceling the food intake reduction effect produced by various peripheral biological satiety signals (including cholecystokinin, exendin-4 (glucagon-like peptide-1 receptor agonist), the amylin, and gastric mechanical dilatation [189]. This discovery not only underscores the synergistic regulatory role of GHS-R1a and OX1R in feeding behavior, but also provides potential targets for developing novel therapeutic strategies against metabolic disorders such as obesity. The article further highlights the shared regulatory influence of GHS-R1a and OX1R on dietary intake.

The discovery of the GHS-R1a/OX1R heterodimer and its unique signal transduction characteristics has opened new avenues for research into GPCR dimerization. Its mechanism of promoting hippocampal neuronal proliferation via the Gαs pathway may offer novel therapeutic approaches for neurodegenerative diseases or cognitive impairments. Nevertheless, the current understanding of this dimer’s physiological functions in vivo and its specific role in disease remains limited. Future research must further investigate its regulatory mechanisms under physiological and pathological conditions, alongside its potential clinical value for targeted interventions.

### 6.7. GHS-R1a/CB1R Heterodimer

Centrally enriched CB1R colocalizes with GHS-R1a in multiple neuronal subpopulations, forming functional heterodimers that synergistically amplify GHS-R1a-mediated Ca^2+^ signaling, potentiate ghrelin-induced orexigenic effects, and are upregulated under high-fat diet-induced metabolic dysfunction.

In 2021, the GHS-R1a/CB1R heterodimer was discovered [48]. CB1R is a G protein-coupled receptor that is highly expressed in the brain and is involved in key regulatory processes such as energy balance, appetite control, and reward [190,191,192]. Recent studies have revealed a significant overlap between GHS-R1a+ and CB1R+ signaling in specific neuronal subpopulations within the cerebral cortex, hippocampus, amygdala, and VTA [193,194,195]. Moreover, CB1R and GHS-R1a exhibit close functional associations [104,196]. Studies have demonstrated that in non-mammalian vertebrates, the GHS-R1a and the CB1R exhibit a significant synergistic mechanism in the regulation of feeding behavior. Specifically, ICV co-administration of ghrelin and a CB1R antagonist into the diencephalon of teleost fish revealed that the CB1 pan-antagonist completely abolished ghrelin-induced hyperphagia and upregulation of NPY mRNA expression [197]. Ghrelin can induce appetite-promoting effects in VTA dopamine neurons, a mechanism that partly involves CB1R-mediated signal transduction [193]. The ghrelin/GHS-R1a system plays a significant role in the rewarding and reinforcing effects of CB1R agonists [195]. These findings suggest that the integration of GHS-R1a and CB1R signaling pathways may serve as a neurobiological basis for appetite promotion. Could these functional interactions be related to the formation of GHS-R1a/CB1R heterodimer? In 2021, Lillo et al. demonstrated that GHS-R1a and CB1R form heterodimers at the level of HEK293 cells and mouse striatal medium spiny neurons using BRET and PLA. This complex not only modifies the Gi signaling of CB1R but also enhances ghrelin-mediated G_q_/Ca^2+^ signaling by 140% [48]. However, the author has not provided us with a list of the specific signaling pathways. Its expression is significantly upregulated in striatal neurons of high-fat diet model mice, suggesting important physiological and pathological significance in energy balance and feeding regulation. This provides affirmative answers to the above questions. These findings may open new avenues for therapeutic interventions in metabolic disorders and obesity.

## 7. Conclusions

**Figure 5:**
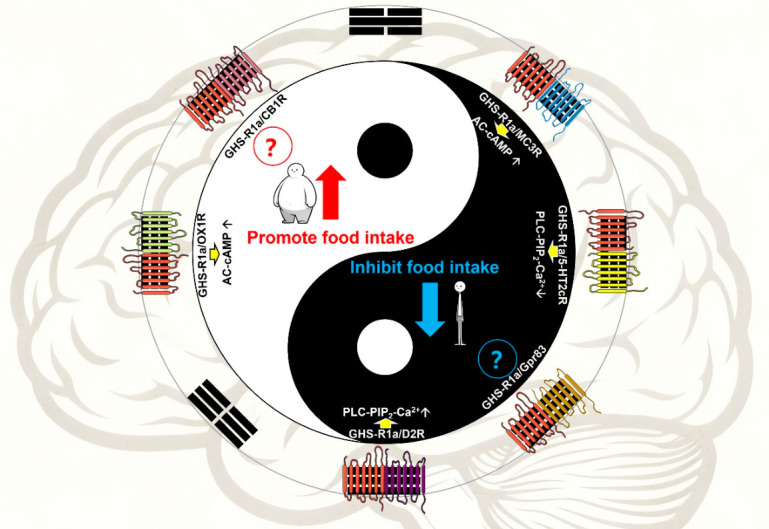
The Yin–Yang of appetite: how GHS-R1a “Dinner-Date” partners tilt the scale. Different colors are used to distinguish the different GHS-R1a heterodimer partners shown in the diagram. The black side shows duos that make you eat more (GHS-R1a/OX1R and GHS-R1a/CB1R); the white side shows duos that make you eat less (GHS-R1a/MC3R, GHS-R1a/5-HT2cR, GHS-R1a/D2R and GHS-R1a/Gpr83). Arrows = “boost” or “brake,” showing a simple snapshot of the push and pull inside your brain when these receptor couples form.

Following an investigation of the above heterodimers of GHS-R1a, it is not difficult to find that although the other GPCR which forms a heterodimer with GHS-R1a is closely related to energy regulation, it exhibits different physiological functions for different heterodimers. For energy regulation, it can be divided into two categories: one is related to promoting food intake, which can be used to treat anorexia; this applies to the above-mentioned GHS-R1a/CB1R and GHS-R1a/OX1R heterodimer. The heterodimer of GHS-R1a may play a more significant role after the introduction of this combination due to the similar functions of the two GPCRs that form the dimer. The other category relates to the negative regulation of energy metabolism, and this function is performed by the GHS-R1a/MC3R heterodimer, GHS-R1a/5-HT2cR heterodimer, GHS-R1a/Gpr83 heterodimer and GHS-R1a/D2R heterodimer. These dimers may become potential targets for the treatment of obesity, because silencing GPCR will greatly weaken ghrelin’s appetite-promoting function (Figure 5).

At present, nearly 30% of the medical drugs in the market target GPCRs [198]. The drugs based on GPCRs monomer have many side effects, especially for the targeted treatment of anorexia or obesity. The discovery of ghrelin, the only known circulating orexin, and the heterodimer its of its receptor GHS-R1a has not only increased the drug’s target, but also increased tissue selectivity because of its differential expression, enhanced the specificity of ligand action and reduced the non-ideal side effects.

## Data Availability

No new data were created or analyzed in this study. Data sharing is not applicable.

## Abbreviations

5-HT2cR: 5-hydroxytryptamine 2c receptor; AC: Adenylyl cyclase; ACTH: Adrenocorticotrophic hormone; AgRP: Agouti-related peptide; AMPK: Adenosine monophosphate-activated protein kinase; BiFC: Bimolecular fluorescence complementation; BiLC: Bimolecular luminescence complementation; BRET: Bioluminescence resonance energy transfer; cAMP: Cyclic adenosine monophosphate; CB1R: Cannabinoid receptor 1; Co-IP: Co-immunoprecipitation; D2R: Dopamine 2 receptor; DAG: Diacylglycerol; ECL: Extracellular loop; FRET: Fluorescence resonance energy transfer; GHS-R1a: Growth hormone-releasing hormone receptor 1a; GPCR: G protein-coupled receptor; Gpr83: G protein-coupled receptor 83; GST: Glutathione-s-transferase; GDP: Guanosine diphosphate; GTP: Guanosine triphosphate; ICL: Intracellular loop; ICV: intracerebroventricular; IP3: Inositol triphosphate; KO: Knockout; LEAP2:Liver-expressed antimicrobial peptide 2; MC3R: Melanocortin 3 receptor; MSH: Melanocyte stimulating hormone; NanoBiT: NanoLuc binary technology; NPY: Neuropeptide Y; OX1R: Orexin receptor 1; PI3K: Phosphatidylinositol 3 kinase; PIP2: Phosphatidylinositol-4,5-diphosphate; PKA: Protein kinase A; PKC: Protein kinase C; PLC: Phosphatidylinositol-specific phospholipase C; PLA: Proximity ligation assay; POMC: Proopiomelanocortin; TM: Transmembrane domain; VTA: Ventral tegmental area.
